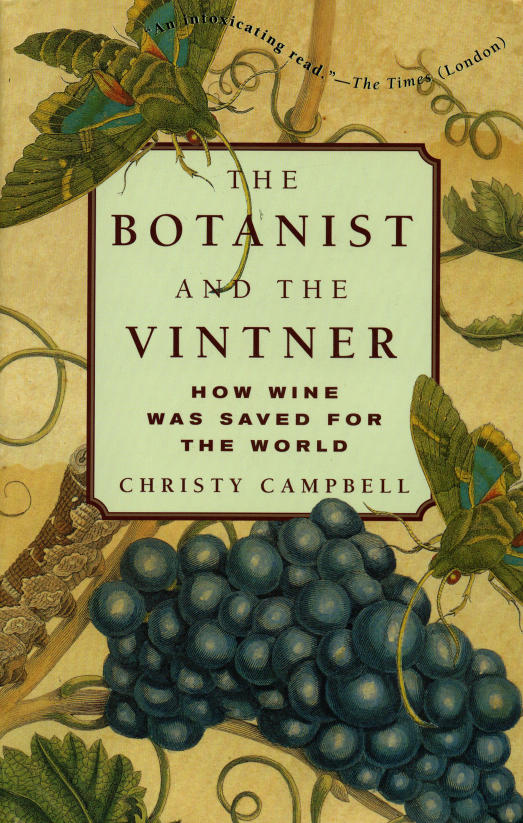# Sour Grapes

**DOI:** 10.1371/journal.pbio.0040102

**Published:** 2006-04-11

**Authors:** William Foster

A phids have never enjoyed a particularly good press. Their huddled masses rain excrement on our parked cars and bring disease and destruction to the crops and gardens of almost all temperate regions. But in the aphid rogues' gallery, one species has pride of place, the arch villain of them all—the grape phylloxera, which practically destroyed the French wine industry in the 19th century. Christy Campbell's book tells the epic story of how this insect was eventually defeated. It is a tale worth telling because this was an immensely important episode in ecological history, indeed a classic of invasion biology, with lessons that resonate for us today as we face mounting threats from the ravages of non-native species.

The bare bones of the story will be familiar to most wine drinkers. Phylloxera was introduced into a French vineyard in the Rhone Valley on the roots of living American vines in the 1860s. French vines, in contrast to most American vines, were very susceptible to the insect, which had evolved with the native American vine species and did not cause them appreciable damage. Phylloxera spread slowly but inexorably across the wine-growing regions of France, reaching the Champagne in 1890, and it eventually conquered all the major wine-growing regions of the world, with the exception of Chile. America provided the cure as well as the cause. American vines, the insects' Trojan horse, were not harmed by the insect, and could be used as rootstocks on which the susceptible European vine species Vitis vinifera could be grafted. This method was amazingly successful and continues to be used to keep the insect at bay. In hindsight the solution may seem obvious, but this book makes clear what an immense struggle was involved. Indeed, Campbell's book illustrates what a difficult, messy, and unpredictable business science can be, and what sheer hard work it is to translate scientific results into action.

The insects themselves are exceptionally difficult to study. They are small (less than 1 mm), they are hidden underground or in galls, and they usually have disappeared by the time the vines have died. The grape phylloxera is not, strictly speaking, an aphid at all, but belongs to Phylloxeridae, which has about 70 species and is part of the sister group to aphids proper. Like aphids, they reproduce asexually for most of the time, but unlike aphids they never bear live young. A single species can have up to 23 different morphs, including the crisply named *neogallicolae–radicicolae*, and they have life cycles of the kind of complexity that has deterred generations of schoolchildren from ever taking up biology. The disentangling of the grape phylloxera's life cycle took years of patient work and was a major barrier to progress.

The story has a large and international cast, with cameos for Darwin and Pasteur, but the leading role is taken by Jules-Emile Planchon, a French Kew Gardens–trained botanist who first established that phylloxera was the disease agent. As well as being an excellent scientist, he was a doughty polemicist, with a vivid turn of phrase. “Facile arguments devoid of facts need only a little puff of rhetoric to seduce the ignoramus,” he wrote in 1874 as the French establishment went into denial about the disease. He was himself capable of great gales of rhetoric. “If Burgundy should be wiped out,” he urged the delegates at a congress in Lyon in 1872 “—along with Bordeaux—one could say that France itself had been overthrown.” The chief supporting actor in the story is Charles Riley, an English-born American entomologist who had fought briefly in the Civil War: a convinced Darwinian, he quickly understood why American but not European vine species were resistant to the insect. Both men were empiricists and happy to get their feet dirty: Riley poured scorn on entomologists who were “too much absorbed in closet studies to make the proper field observations.”

Unmasking the culprit was only the beginning of the battle. The French government's offer of a prize of 300,000 francs for a practical cure provoked a torrent of remedies. These included snail slime, lard, volcanic ash from Pompeii, and living toads immured in the soil. Urine was a particular favourite: human, animal, dried, or direct from source. Schoolboys in the Beaujolais were conducted twice daily from their classes to urinate on the vines—no doubt a welcome break from tussling with the subjunctive. Some methods did have a measure of success. These included drowning the pests by localized flooding and growing the vines in sand, which killed the insects either by denying them enough air space or, a possibility not mentioned by Campbell, by scratching their delicate waterproof cuticles. Carbon bisulphide was also effective on a local scale, and chemical warfare was waged on the insect as armies of *piqueurs* travelled the wine-growing areas injecting the substance into the ground with huge metal syringes.

The biologists realized that salvation would come only by planting resistant American vines, but progress was slow and erratic. Wine produced direct from the American species disgusted the French palate and was derided for its foxy taste—the *goût de renard*. It is immensely irritating to discover, right at the end of the book, that American wines now titillate the more discriminating sommeliers who enthuse about their “notes of jammy black fruits with touches of sweet spices—the complex and subtly nuanced nose, the curvature of attack finished by a velvety completeness …” This nonsense cut no ice with the wine drinkers of the 19th century, and the only solution was to graft the French vines onto American rootstocks and hope that the foxiness did not sneak its way up into the fruit. At a congress in 1881, it was pronounced that the grafted vines seemed to produce acceptable wine, and large-scale replanting got under way. It was a colossal task and there were major setbacks. One particular problem was that very few of the American species could cope with the calcareous soils of many of the famous wine-growing regions. Eventually the battle was won. Virtually all the wine we drink today comes from grafted rootstocks. But the war smoulders on: new aphid biotypes appeared in the 1980s that were able to feed on Californian rootstocks, and research on developing genetically modified vines is now in full swing.

The original UK version of the book was entitled *Phylloxera: How Wine Was Saved for the World*. For this version, the publishers have evidently decided that a four-syllable word is too taxing for the American market and have instead grafted *The Botanist and the Vintner* onto this title. This is cumbersome and meretricious: it suggests that the book is about the personal relationship between two individuals, discussing the latest grafting technique between bouts of mildly unconventional lovemaking. Campbell writes engagingly, although the timelines do occasionally get knotted. The first chapter has a fair bit of *Reader's Digest*–style imaginative reconstruction: “Marshall P. Wilder swirled the dark red liquid, sipped delicately and dabbed his enormous moustache with a linen napkin. There was a grunt of approval.” Fortunately, the author tires of this long before we do, and there is no more grunting. Wine lovers and biologists will enjoy this salutary tale of an ecological battle that rumbles on to the present day, and aphid lovers will thrill to this tribute to the power of these minute but mighty insects.

**Figure:**